# Modified clock mapping biopsy sec. Della Corte-Bifulco in the preoperative assessment of excisional surgery for vulvar Paget’s disease

**DOI:** 10.3389/pore.2024.1611803

**Published:** 2024-06-24

**Authors:** Luigi Della Corte, Mario Ascione, Giuseppe Bifulco

**Affiliations:** ^1^ Department of Neuroscience, Reproductive Sciences and Dentistry, School of Medicine, University of Naples Federico II, Naples, Italy; ^2^ Department of Public Health, University of Naples Federico II, Naples, Italy

**Keywords:** gynecological cancer, extramammary Paget’s disease of the vulva, vulvar malignant lesion, preoperative biopsy, new technique

## Abstract

We have developed a biopsy technique aimed at preoperative evaluating the extent of Paget’s vulvar disease in order to plan subsequent radical vulvar surgery. The aim is to find all possible lesion sites that are not visible macroscopically, to obtain a clear evaluation of the disease spread and to tailor the radical surgical procedure to remove even microscopic lesions, avoiding recurrences and excessively destructive surgery, adopting as conservative an approach as possible. We used this procedure for the first time to establish the radicality of the surgical intervention in a 68-year-old patient initially suffering from a single invasive vulvar Paget’s lesion.

## Introduction

Extramammary Paget’s disease of the vulva (EMPDV) is a rare non-squamous skin neoplasm, which is defined as an intraepithelial neoplasm of epithelial origin that expresses glandular characteristics. Histologically, it is characterized by the presence of large cells with prominent cytoplasm, called Paget cells [1, 2]. Vulvar localization accounts for 65% of cases of extramammary Paget’s disease but constitutes only 1%–2% of genital neoplasms [3]. Due to its rarity and unclear clinical presentation, EMPDV is often diagnosed late, when the lesions are already quite extensive [4]. Although the lesions generally follow a slow and indolent evolution, in some cases the pathology may spread deeply, into the lymph nodes, and more rarely by the hematogenous route [5]. In the majority of cases of EMPDV, a discordance is established between the macroscopic and histological margins of the lesion: in fact, lesions may skip, be multifocal, or develop asymmetrically, which makes achieving negative surgical margins challenging [6]. Therefore, the perilesional satellite areas, which are macroscopically and clinically free, appear to be the site of disease on histopathological examination. For this reason, although surgical excision is the gold standard in the treatment of EMPDV, it is often insufficient in the evaluation of clinically occult lesions, leading to recurrence and the need for multiple excisional interventions with sometimes destructive and/or mutilating outcomes [7–9]. Other surgical techniques have been described in the literature aimed at pre-operatively evaluating the extent of the surface to be removed in order to minimize the possibility of recurrence. The Japanese experience had already highlighted how a biopsy mapping of the disease was superior over MMS (Mohs micrographic surgery), which instead aimed to practice a progressive excision of clinically evident lesions with an inevitably higher rate of recurrence [10]. Subsequently, Garganese et al. have proposed a “clock mapping” technique [11], which consists of performing multiple preoperative vulvovaginal biopsies at different sites both in the superficial vulvo-perineal area and in the central deep vaginal area, as follows:

- within the visible lesion, corresponding to any suspiciously thickened area;
- outside the visible lesion, at the level of each position of the clock, at three radial points defined as point A (corresponding to the margin of the lesion) and points B and C, respectively, which are at a distance of 2 and 4 cm from the edges of the lesion;
- at three vaginal levels for each cardinal point, i.e., at the vestibule and a distance of 2 and 4 cm from it.

However, despite the evidence reported in the literature regarding the prediction of the invasiveness and extension of EMPDV, this surgical strategy consists of 48 biopsy samples (36 in the vulvar region and 12 in the vaginal region) and, therefore, requires the execution of a complex and time-consuming surgical procedure. From these considerations, we had the intuition to develop a new biopsy sampling technique to be proposed in the proper evaluation of EMPDV that is inspired by the models described so far in the literature but provides innovative and simplifying elements while maintaining adequate diagnostic efficiency.

## Description of the surgical technique

**FIGURE 1:**
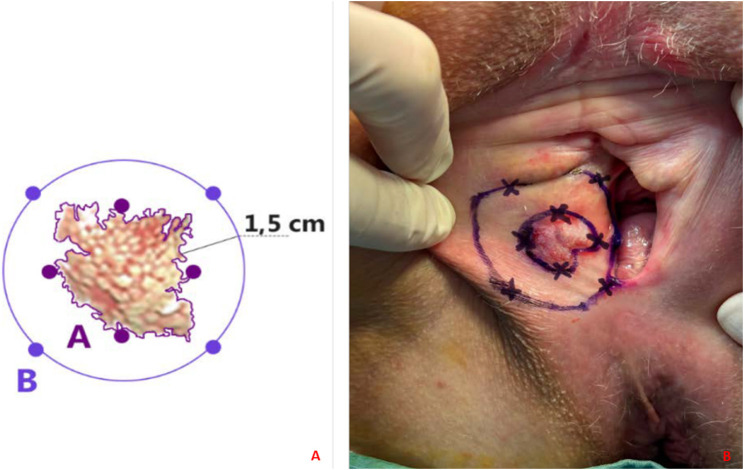
**(A, B)** Modified clock mapping technique. **(A)** A graphic representation of the sites where the biopsy should ideally be performed. The clinically evident edges of the lesion constitute perimeter A. Perimeter B is drawn 1.5 cm away from A; **(B)** *In vivo* application of clock mapping.

Modified clock mapping biopsy sec. Della Corte-Bifulco consists of identifying the macroscopically evident lesion in the vulvar region, which represents the center of an imaginary clock. Biopsy samples are taken along two routes: perimeter A, corresponding to the margin of the clinically evident lesion, and perimeter B, 1.5 cm away from the first. Biopsy sampling is carried out as follows:

• Along perimeter A, four biopsy samples are taken at the four cardinal points, i.e., at 3, 6, 9, and 12 o’clock;
• Along perimeter B, four biopsy samples are obtained in such a way that they are misaligned with respect to the first ones. Specifically, the biopsy is taken at the midpoint between 1 and 2 h, then between 4 and 5 h, between 7 and 8 h, and finally between 10 and 11 h (Figure 1A).

Each biopsy sample must have an extension of at least 1 cm in diameter.

## Application

A 68-year-old woman was referred to us after a vulvar biopsy was performed at another institution with a histological diagnosis of invasive EMPDV. She had been complaining of chronic itching for approximately 6 months. She had undergone bilateral mammography, colonoscopy, and urine cytology to exclude other EMPD localizations. The lesion appeared erythematous and ulcerated with a minute whitish nodule medially on the middle third of the right major labia, measuring 3 cm and 2 cm from the midline. Its edges appeared blurred. Preoperative Magnetic Resonance Imaging, and 18F-2-deoxy-2-fluoro-D-glucose Positron Emission Tomography were negative. Therefore, we decided to apply the “modified clock mapping” surgical technique in order to better understand its spread and to establish the tissue excision margins (Figure 1B). As described in the literature, this disease is characterized by the presence of “skip lesions,” sometimes not macroscopically evident but responsible for recurrence. Thanks to our strategy, the invasive EMPDV was histologically confirmed at 9 o’clock (perimeter A) and, in addition, the pathologist discovered the presence of intraepithelial disease foci, 1.5 cm away from the main lesion, in particular at 7 o’clock (perimeter B) at the level of the posterior fourchette.

**FIGURE 2:**
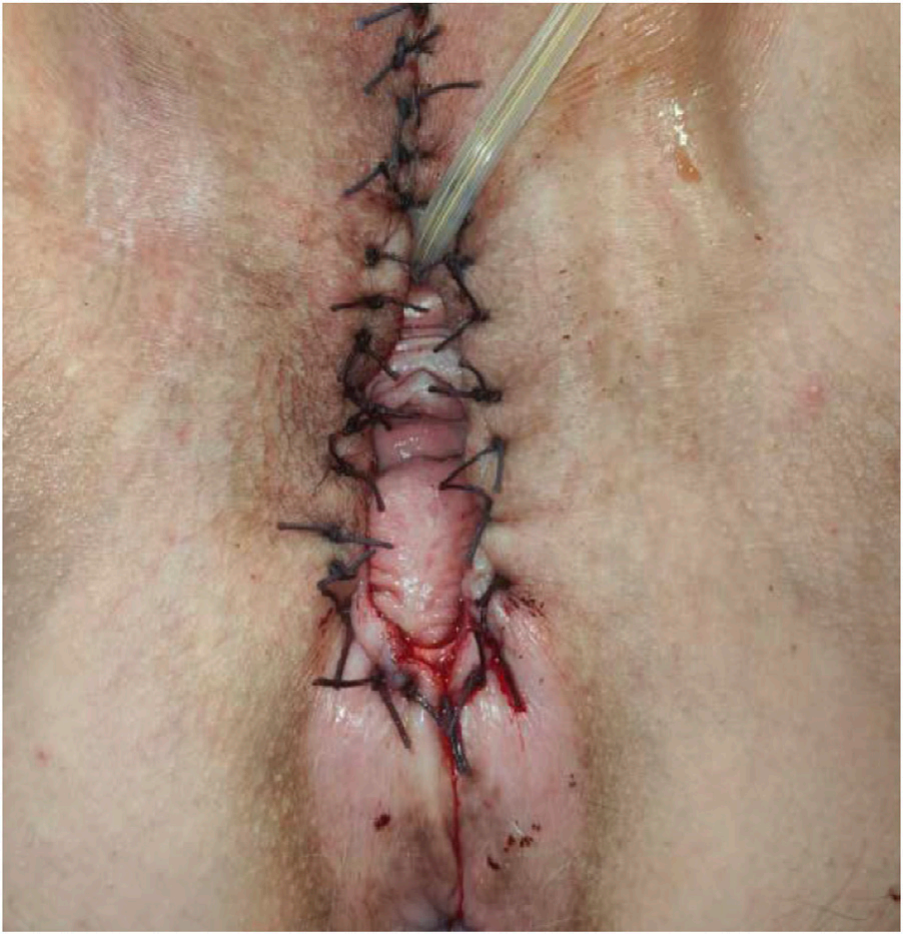
Final result of radical total vulvectomy.

Finally, after detailed counseling and obtaining written informed consent, the surgeon (L.D.C.) performed a total radical vulvectomy with an ipsilateral inguinofemoral sentinel lymph node biopsy (Figure 2). Specifically, technetium(99) m-labeled microcolloid was injected intradermally at four locations around the tumor 5 h before the planned procedure. Dynamic and static images were recorded using a gamma camera. The right SLN location was marked on the overlying skin. In the operating room, the SLN was identified at the beginning of the procedure using a handheld gamma-detection probe. Regarding the vulvar surgical time, the lesion was removed with 1 cm of free margin outside the 7 o’clock point.

**FIGURE 3:**
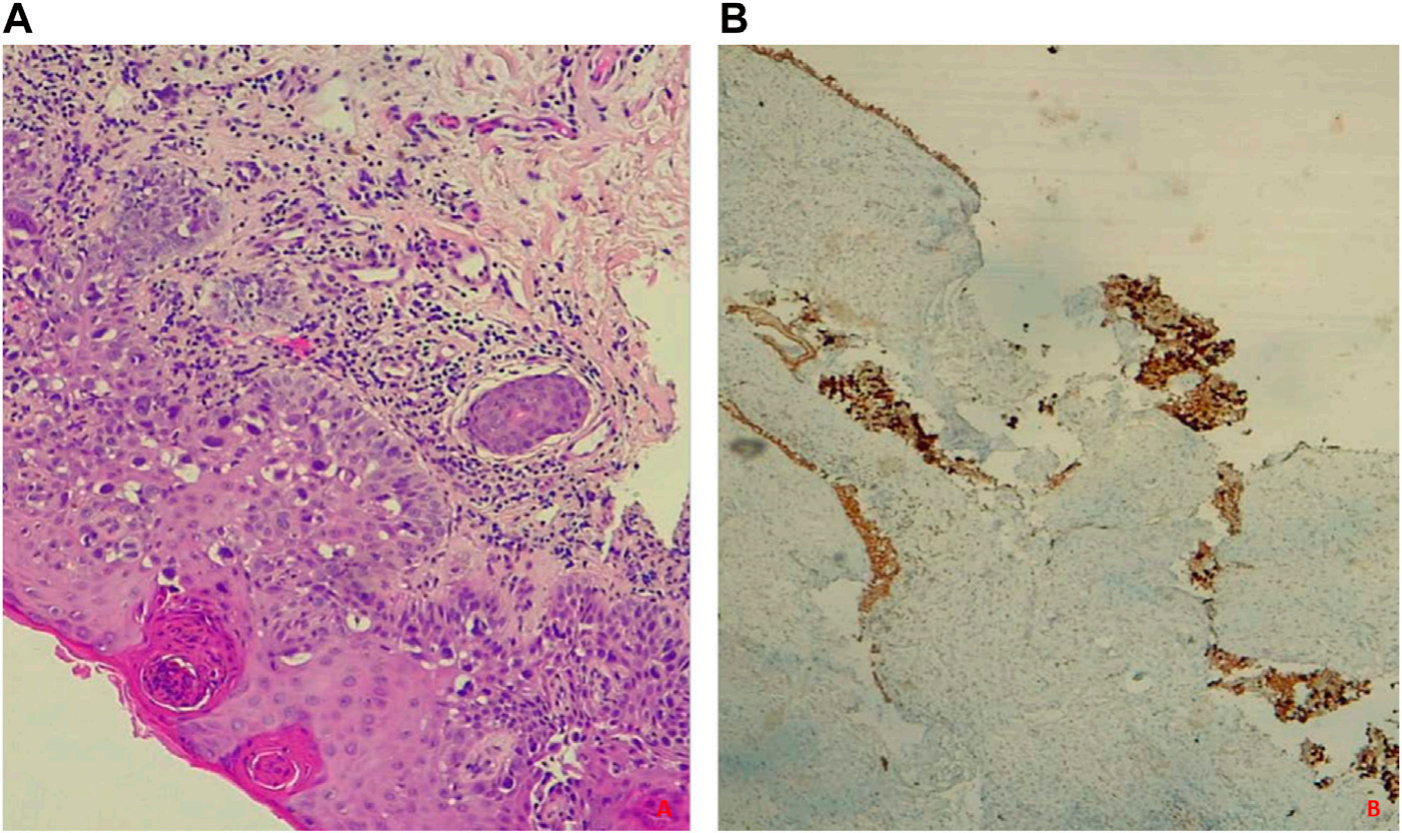
**(A, B)** Intraepithelial Paget’s disease. **(A)** Hematoxylin-eosin staining shows atypical, polygonal, large glandular cells with abundant vacuolated cytoplasm, hyperchromic nuclei, and evident nucleoli resembling Paget’s cells; **(B)** GATA3 Immunohistochemical Staining is positive.

The histological examination confirmed the dual vulvar diagnosis of invasive and non-invasive EMPDV with 1 cm of healthy surgical margin (Figures 3A, B), while the sentinel lymph node was negative. No early or late postoperative complications were recorded. After 6 months of follow-up, no recurrence has been described and the patient is in good health.

## Conclusion

A high rate of invasiveness and recurrence characterizes EMPDV. Frequently, it manifests as skip lesions, which are not always clinically evident, as in our case. The “modified clock mapping” technique is feasible and safe for the preoperative evaluation of vulvar Paget’s disease and avoids an excessive and sometimes unnecessary number of vulvar biopsies without the risk of underdiagnosis. It has allowed us to identify an area of satellite disease that otherwise would have remained unrecognized, leading to recurrence: this aspect is particularly important because it would avoid conservative treatment in the case of unknown lesions far from macroscopically evident ones, but also demolitive surgery when not necessary. Moreover, it could have a positive impact on the quality of life of the patients and a better aesthetic result. Further studies on a larger sample of patients are needed to estimate the impact of this surgical strategy in terms of recurrence-free survival in women with EMPDV.

## Data Availability

The raw data supporting the conclusion of this article will be made available by the authors, without undue reservation.
